# Interferon-beta enhances sensitivity to gemcitabine in pancreatic cancer

**DOI:** 10.1186/s12885-020-07420-0

**Published:** 2020-09-23

**Authors:** Amber Blaauboer, Stephanie Booy, Peter M. van Koetsveld, Bas Karels, Fadime Dogan, Suzanne van Zwienen, Casper H. J. van Eijck, Leo J. Hofland

**Affiliations:** 1 Department of Surgery, Erasmus Medical Center, Room Ee-514, Doctor Molewaterplein 40, 3015 GD Rotterdam, The Netherlands; 2 Department of Internal Medicine, Division of Endocrinology, Erasmus Medical Center, Rotterdam, The Netherlands

**Keywords:** Pancreatic cancer, Interferon-beta, Gemcitabine, Chemosensitising effect, Drug transporters

## Abstract

**Background:**

Adjuvant gemcitabine for pancreatic cancer has limited efficacy in the clinical setting. Impaired drug metabolism is associated with treatment resistance. We aimed to evaluate the chemosensitising effect of interferon-beta (IFN-β)*.*

**Methods:**

BxPC-3, CFPAC-1, and Panc-1 cells were pre-treated with IFN-β followed by gemcitabine monotherapy. The effect on cell growth, colony formation, and cell cycle was determined. RT-qPCR was used to measure gene expression. BxPC-3 cells were used in a heterotopic subcutaneous mouse model.

**Results:**

IFN-β increased sensitivity to gemcitabine (4-, 7.7-, and 1.7-fold EC_50_ decrease in BxPC-3, CFPAC-1, and Panc-1, respectively; all *P* < 0.001). Findings were confirmed when assessing colony formation. The percentage of cells in the S-phase was significantly increased after IFN-β treatment only in BxPC-3 and CFPAC-1 by 12 and 7%, respectively (*p* < 0.001 and *p* < 0.05, respectively). Thereby, IFN-β upregulated expression of the drug transporters *SLC28A1* in BxPC-3 (252%) and *SLC28A3* in BxPC-3 (127%) and CFPAC-1 (223%) (all *p* < 0.001). In vivo, combination therapy reduced tumor volume with 45% (*P* = 0.01). Both ex vivo and in vivo data demonstrate a significant reduction in the number of proliferating cells, whereas apoptosis was increased.

**Conclusions:**

For the first time, we validated the chemosensitising effects of IFN-β when combined with gemcitabine in vitro, ex vivo, and in vivo. This was driven by cell cycle modulation and associated with an upregulation of genes involving intracellular uptake of gemcitabine. The use of IFN-β in combination with gemcitabine seems promising in patients with pancreatic cancer and needs to be further explored.

## Background

Pancreatic cancer is a highly malignant disease, with an estimated 5-years survival of 9% [1]. At diagnosis, approximately 15% of the patients have resectable disease (stage I or II), which indicates surgery followed by systemic therapy [2]. The CONKO-001 trial is the first randomized controlled trial in pancreatic cancer that reported longer overall survival (OS) rates in patients treated with adjuvant gemcitabine compared with observation alone. However, the median OS with this regimen is still only 22 months [3]. Recently, Conroy et al. demonstrated a significant survival benefit with modified FOLFIRINOX compared to gemcitabine alone (PRODIGE-24 trial) in highly selected patients [4]. In addition, new chemotherapeutic strategies in combination with gemcitabine are currently explored and also improved the prognosis of pancreatic cancer [4, 5]. However, these new regimens are associated with severe toxicity in comparison with gemcitabine alone. Since the majority of patients are elderly or have serious comorbidity, gemcitabine is often the only therapeutic option. The resistance of this chemotherapeutic is still a major impediment of successful systemic treatment for patients diagnosed with pancreatic cancer.

The limited efficacy of gemcitabine has been associated with impaired drug metabolism, hindering its intracellular uptake and activation [6]. Gemcitabine, 2′,2′-difluoro 2′-deoxycytidine (dFdC), is a hydrophilic pro-drug that requires cellular uptake and intracellular phosphorylation. Several transporters have been identified, but its major uptake is through hENT1, hCNT1, and hCNT3 (encoded by *SLC29A1*, *SLC28A1*, and *SLC28A3*, respectively) [7]. Expression levels of these transporters correlate with gemcitabine sensitivity and OS in pancreatic cancer, making them good predictive markers [8, 9].

Once inside the cell, gemcitabine requires a serial of phosphorylation by multiple kinases to become pharmacologically active. The cytotoxic activity of gemcitabine is a result of several inhibitory actions on DNA synthesis. Incorporation into new DNA strands as the cell replicates is the most likely mechanism by which gemcitabine causes cell death. This incorporation creates an irreparable error (masked chain termination) that leads to inhibition of DNA synthesis, and thus apoptosis.

Deoxycytidine kinase (dCK) catalyzes the initial and rate-limiting monophosphorylation of gemcitabine. However, the majority (~ 90%) of intracellular gemcitabine is directly inactivated by deamination by cytidine deaminase (CDA) to form 2′2’ difluorodeoxyuridine (dFdU), which is subsequently degraded and excreted out of the cell [7]. Thus, deficiency in dCK is a major contributor to gemcitabine resistance, while upregulation of CDA has been shown to confer gemcitabine resistance in pancreatic cancer [10, 11].

Prior studies have proposed a potential role for type I IFNs (IFN-α and -β) in combination with gemcitabine in the treatment of pancreatic cancer. IFN-α and -β were originally identified as immunomodulatory cytokines, due to their antiviral activity. Further characterization of their biological effect revealed a wide range of potential anti-tumor effects, e.g. inhibition of cell proliferation, induction of apoptosis, and cell cycle arrest. Importantly, type I IFNs have been shown to sensitize cancer cells to chemo- and radiotherapy [12–14].

Both IFN-α and -β interact with the type I IFN receptor complex, which initiates the JAK/STAT downstream pathway, resulting in subsequent transcription of interferon stimulated genes (ISGs). These ISGs encode for numerous proteins that initiates different IFN activities, including anti-tumor effects, immunoregulatory effects, and other host effects [15].

While prior research has primarily focused on the anti-tumor activities of IFN-α, studies have reported that the direct anti-tumor effects of IFN-β are much stronger, and elicited at much lower concentrations, as compared to IFN-α [16, 17]. However, there are no animal or clinical studies that investigated the use of concomitant adjuvant IFN-β therapy in the treatment of pancreatic cancer.

In the present study, we aimed to further explore the potential chemosensitising effect of IFN-β and studied the mechanism of action in three human pancreatic cancer cells lines. For the first time, we demonstrate the interaction between IFN-β and the expression of genes involved in gemcitabine transport and metabolism. In addition, we evaluated the effects of IFN-β and gemcitabine in a pancreatic cancer xenograft tissue slice model. Finally, we confirmed the anti-tumor effect of IFN-β in combination with gemcitabine in a heterotopic pancreatic cancer mouse model.

## Methods

### Cell culture and compounds

Three human pancreatic cancer cell lines (BxPC-3, CFPAC-1, and Panc-1) were used and obtained from American Type Culture Collection (Rockville, MD, USA). Short tandem repeat profiling using a Powerplex Kit (Promega, Leiden, the Netherlands) of the cells gave consistent results with the ATCC database. Cells were confirmed as mycoplasma-free. Culture conditions were described in detail previously [18]. Human recombinant IFN-β-1a (Rebif, Rockland, MA, USA) and gemcitabine (Sigma-Aldrich, Zwijndrecht, the Netherlands) stock solutions, prepared in H_2_O, were stored at 4 °C. After trypsinization, cells were plated at the appropriate density in order to obtain 80% confluency at the end of the experiment. The next day, incubations with the indicated compounds were initiated and control cells were vehicle treated. For seven-day experiments, both drug compounds and medium were refreshed after 3 days. All cell culture experiments were carried out at least twice in quadruplicate.

### Cell proliferation assay

Treatment effects were assessed on DNA amount as measure of cell number. After treatment, media were removed and plates were stored at − 20 °C until DNA measurement. Measurement of total DNA was performed with the bisbenzimide fluorescent dye (Hoechst 33258, Sigma-Aldrich, Zwijndrecht, the Netherlands) as previously described [19].

### Colony-forming assay

Plates were coated with 1 mL poly-L-lysine (10 μg/mL), where after 1500, 200, or 300 cells were plated in a 6 wells plate for BxPC-3, CFPAC-1, and Panc-1, respectively. After 1 day, drug treatment was initiated. After treatment, media were removed and refreshed without drugs. When colonies contained at least 50 cells (2 weeks for all cell lines), cells were washed and stained with haematoxylin. Amount and size of colonies was measured using MultiImage light cabinet (Alpha Innotech) and the ImageJ software. Plating efficiency (PE) was calculated as the mean number of colonies/number of plated cells for control cultures not exposed to drugs. Surviving fraction was calculated as the mean number of colonies/(number of inoculated cells x PE).

### Cell cycle analysis

After treatment, cells were harvested, washed with NaCl, fixed with ice-cold 70% EtOH, and stored at − 20 °C until analysis. Analyses were performed using the Muse® Cell Cycle Assay Kit utilizing Muse™ Cell Analyser (Merck Millipore, Amsterdam, the Netherlands).

### Real-time quantitative PCR

Total RNA was isolated using the High Pure RNA Purification Kit (Roche). Yield and purity were assessed with the nanodrop. cDNA was synthesized from mRNA template by reversed transcription. To synthesize cDNA, 500 ng mRNA template was added to 40 μL Super RT buffer (Thermofisher Scientific, the Netherlands) containing 40 nmol dNTP, 20 U RNA’sin, 15 ng oligo-dT, 4 U Super RT. After 1-h incubation at 40 °C, cDNA was five times diluted. 7.5 μL TaqMan Universal PCR Master Mix (Applied Biosystems) was mixed with concentrations of the used primers and probes, with 5 μL cDNA template. The RT-PCR reaction was performed using the 7900HT Fast Real-Time PCR System. Three housekeeping genes (*HPRT*, *β-actin,* and *GUSB*) were used to normalize mRNA levels using the Vandesompele method (Thermo Fisher Scientific, Breda, the Netherlands) (Table S1) [20].

### Animals and heterotopic injection of BxPC-3 tumor cells

Male athymic Balb/C nude mice (commercially obtained from Harlan laboratories, UK ltd) of 8 weeks old, were used and kept in a barrier facility under HEPA filtration. BxPC-3 cells (1 × 10^6^/100 μl PBS) were subcutaneously injected at the flank after which the mice were randomized into four groups (*n* = 8 each). A separate group (*n* = 4), which did not receive any treatment, was used for tissue slice experiments. All mouse experiments were controlled by the animal welfare committee (IvD) of the Erasmus Medical Center and approved by the national central committee of animal experiments (CCD) under the protocol number 105–12-52, in accordance with the Dutch Act on Animal Experimentation and EU Directive 2010/63/EU.

### Therapy and assessment of tumor size

Tumor size and body weight was measured twice weekly. Tumor volumes were calculated as (lengthxwidth)^1.5^x(π/6). Treatment was started when tumor volumes reached ~150mm^3^. Mice in the control group and IFN-β group received five times a week, on consecutive days, an i.p. injection of 100 μl of 0.9% NaCl or 1.5 × 10^5^ IU of IFN-β. Mice in the gemcitabine group received on day 2 and 4 40 mg/kg gemcitabine i.p. Mice in the combination group recieved five times a week, on consecutive days, an injection of 1.5 × 10^5^ IU of IFN-β i.p. and, on day 2 and 4, an 40 mg/kg gemcitabine i.p.

### Necropsy procedures

Mice were sacrificed by cervical dislocation under isoflurane anesthesia after 4 weeks of treatment, when tumor volume reached 2000mm^3^ (1500mm^3^ for tumors of mice used in the tissue slice experiments), or when the wellbeing (i.e. weight loss, lethargy, tumor ulceration) of the mice could no longer be maintained.

During necropsy, tumors were resected and tumor weight and volume were measured. Tumors were divided into three parts and subsequently snap frozen in liquid nitrogen, embedded in Tissue-Tek (Sakura Finetek, Zoeterwoude, the Netherlands) for cryosectioning and fixed in freshly prepared 4% formaldehyde solution, and prepared for paraffin sectioning. Tumors were harvested, weighted and fixed in 4% formaldehyde.

### Tissue slicing and slice culture

When tumors reached a volume of 1500 mm^3^, mice were sacrificed and necropsy was performed as described above. After resection, tumors were washed twice with Hanks’ Balanced Salt Solution (HBSS) supplemented with penicillin (1 × 10^5^ U/L), streptomycin (1000 IU/ml) and fungizone (30 μg/ml).

After the vibrocheck (measurement of vertical deflection) was performed, tumor specimens, buffered in ice-cold HBSS, were cut, with stainless steel razor blades, into slices of 200 μm using the Leica vibrating blade microtome VT1000 S (Leica, Wetzlar, Germany). After slicing, samples were washed once more and transferred into six-well multiplates containing 5 ml of culture medium consisting of Dulbecco’s Modified Eagle’s Medium: nutrient mixture F-12 (DMEM/F-12), supplemented with penicillin (1 × 105 U/L), streptomycin (1000 IU/ml) and 10% FCS. Consecutive slices were used for the experiments (minimum of 15 slices per tumor).

Tissue culture plates were placed in a humidified incubator at 5% CO_2_ and continuously shaken (60 rounds/min) at 37 °C up to 4 days post slicing. Media and supplements were obtained from GIBCO Bio-cult Europe (Invitrogen, Breda, The Netherlands). After 24 h, media were refreshed and slices were incubated with IFN-β (100 IU/ml), gemcitabine (1 ng/ml), or with the combination of IFN-β and gemcitabine. After 72 h of incubation, tissue slices were harvested fixed in freshly prepared 4% formaldehyde solution and prepared (in upright position) for standard paraffin sectioning.

### Immunohistochemistry

The formalin fixed and paraffin embedded sections (5 μm thick) were treated for immunohistochemistry as described previously [21]. Briefly, sections were deparaffinised and rehydrated, followed by heat induced epitope retrieval, rinsed (TRIS/Tween 0.5% (pH 8.0)) and blocked (hydrogen peroxide 3% in PBS) for 15 min before incubation with the primary antibodies for Caspase-3 and Ki-67 (both overnight at 4 °C). For negative controls, the primary antibody was omitted. The Dako Real EnVision Detection System kit (Dako Detection System, Dako Denmark, Glostrup, Denmark) was used to visualize the bound antibody after which the slides were counterstained with haematoxylin and coverslipped. The rabbit monoclonal Cleaved Caspase-3 (Asp175) antibody (cell signalling technology, Beverly, MA, USA) was used at a dilution of 1:750. The mouse monoclonal Ki-67 antibody (Dako Detection System) was used at a dilution of 1:400.

### Immunohistochemical analysis

All sections were evaluated and counted by AB using CellProfiler (cell image analysis software) [22]. Apoptosis and cell proliferation were assessed by counting the total number of caspase-3 or Ki-67 positive tumor cells per high-power field (Olympus, Nikon Eclipse E400, HPF × 40 objective). For the analysis, a minimum of 3 HPF were used to evaluate cells with a positive and negative staining.

### Statistical analysis

GraphPad Prism version 3.0 (GraphPad Software) was used for statistical analysis. Non-linear regression curves were used to calculate the half maximal effective concentration (EC_50_) on cell growth. For analysis of the combination therapy, effect of IFN-β was set on 100% and used as control. One-way ANOVA test with Tukey’s multiple comparisons test was used for comparisons among treatment groups. Regarding in vivo experiments, differences between groups were evaluated by the Mann-Whitney t-test. In all analyses, values of *p* < 0.05 were considered as significant. Data are indicated as mean ± SEM, unless specified otherwise.

## Results

### IFN-β and gemcitabine dose-dependently inhibit proliferation in human pancreatic cancer cells

**Fig. 1:**
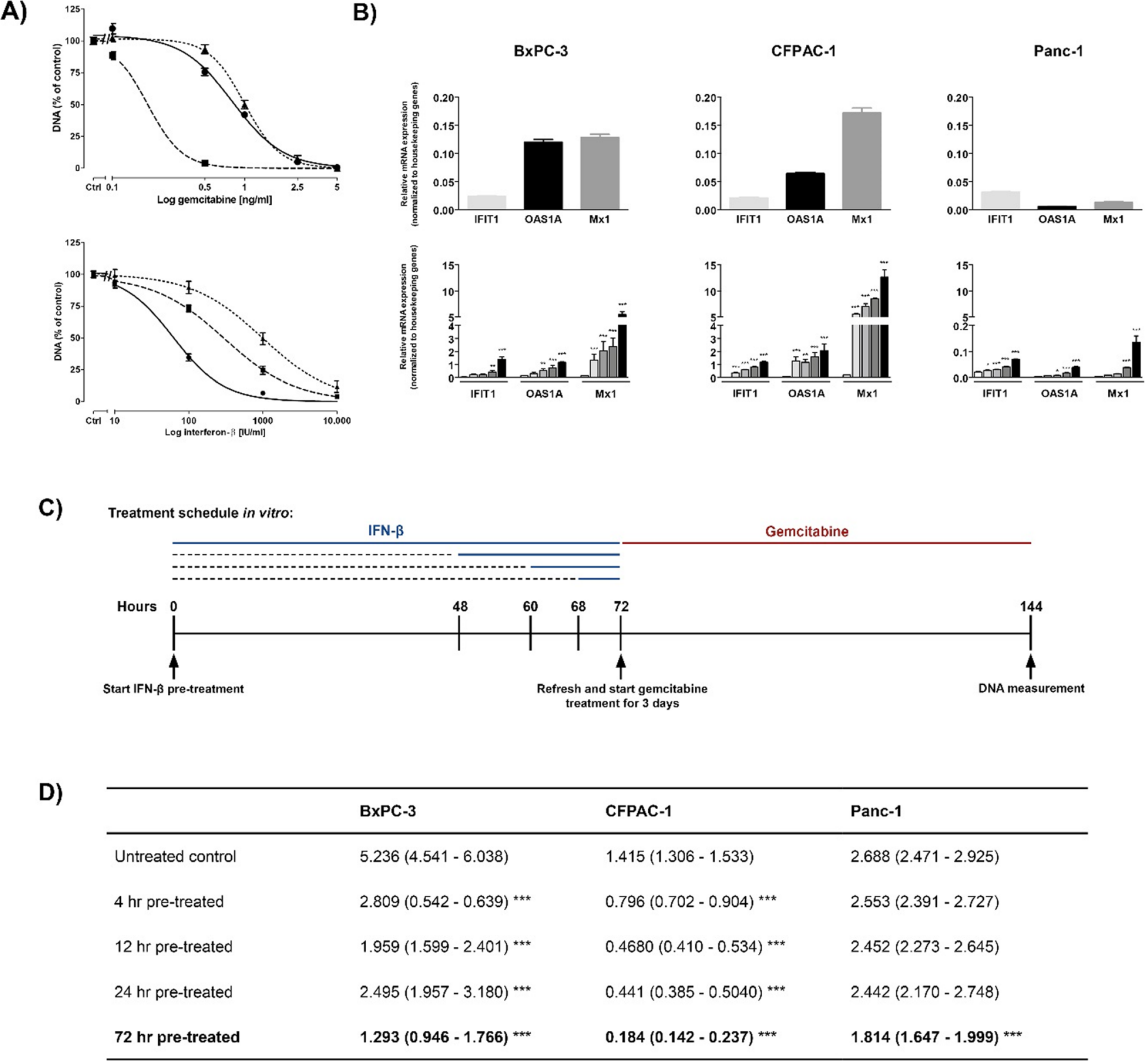
In vitro treatment effects of gemcitabine and IFN-β. **a** Dose response curves of gemcitabine and IFN-β on total DNA amount, as a measure of cell number, in ● BxPC-3, ■ CFPAC-1, and ▲ Panc-1 after 7 days of treatment. **b** Upper panel represents baseline mRNA expression of *IFIT1*, *OAS1A*, and *Mx1* in BxPC-3 (left panel), CFPAC-1 (middle panel), and Panc-1 (right panel); Lower panel represent relative mRNA expression in untreated control cells and after 4 (white bar), 12 (light grey bar), 24 (dark grey bar), or 72 h (black bar) pre-treatment with IFN-β. **c** Experimental design for in vitro experiments. Cell were pre-treated with IFN-β for 4, 12, 24 or 72 h, followed by 72 h gemcitabine monotherapy. **d** EC_50_ values of gemcitabine on cell growth in non-IFN-β pre-treated cells vs IFN-β pre-treated cells. EC_50_ values are presented in nanogram per milliliter (ng/ml, 95% CI). EC_50_ values depicted in bold represent the strongest decrease. Used concentrations IFN-β: 100 IU/ml for BxPC-3 and CFPAC-1, and 1000 IU/ml for Panc-1. Values represent mean ± SEM of at least two independent experiments in quadruplicate and are shown as the percentage of control. *p < 0.05, **p < 0.01, and ***p < 0.001 versus control

To assess the growth inhibitory effect of IFN-β and gemcitabine, cells were treated with increasing concentrations (10–10.000 IU/ml) IFN-β or (0.1–5 ng/ml) gemcitabine during 3 or 7 days. Both drugs inhibited cell growth, measured as DNA amount per well, in a dose- and time dependent manner (Fig. 1a and S1).

After 7 days, IFN-β inhibited cell growth in BxPC-3 at significantly lower concentrations (EC_50_ 62 IU/ml; 95% CI 7.4–66.4) than CFPAC-1 (302 IU/ml; 95% CI 285.6–319.5; *p* < 0.001) and Panc-1 (978 IU/ml; 95% CI 896.8–1067; *p* < 0.001). For gemcitabine, EC_50_ value after 7 days was lower for CFPAC-1 (0.19 ng/ml; 95% CI 0.17–0.2), compared to BxPC-3 (0.81 ng/ml; 95% CI 0.79–0.84; *p* < 0.001) and Panc-1 (1.0 ng/ml; 95% CI 0.98–1.02; *p* < 0.001).

### Time-dependent upregulation of expression of interferon-stimulated genes by IFN-β

Gene expression analysis of three ISGs (*IFIT1*, *OAS1A*, and *Mx1)* was performed after cells were treated for 4, 12, 24 or 72 h with 100 IU/ml (BxPC-3 and CFPAC-1) or 1000 IU/ml (Panc-1) IFN-β. The average baseline expression of all three genes was higher in BxPC-3 and CFPAC-1, compared to Panc-1 (Fig. 1b, upper panel).

All genes were upregulated during IFN-β treatment in a time-dependent manner (Fig. 1b, lower panel). The strongest induction was observed in *Mx1* after 72 h treatment. In BxPC-3 and CFPAC-1, *Mx1* expression was already significantly upregulated after 4 h treatment with IFN-β. In Panc-1, a significant lower increase of all three genes was observed, even though cells were treated with 1000 IU/ml IFN-β.

### IFN-β sensitizes pancreatic cancer cells to gemcitabine treatment

Next, we assessed the potential chemosensitising effect of IFN-β, as indicated by a fold decrease in EC_50_ value of gemcitabine. Cells were pre-treated for 4, 12, 24 or 72 h with 100 IU/ml (BxPC-3 and CFPAC-1) or 1000 IU/ml (Panc-1) IFN-β, followed by 3 days of increasing concentrations (0.1–5 ng/ml) gemcitabine monotherapy (Fig. 1c).

The overall response to gemcitabine was significantly stronger in IFN-β pre-treated cells compared to untreated control cells, even at time points where the effect of IFN-β on cell growth was minimal (Fig. S2).

This chemosensitising effect was most evident when cells were pre-treated for 72 h with IFN-β, as shown by a 4-, 7.7-, and 1.7-fold decrease in EC_50_ value in BxPC-3, CFPAC-1, and Panc-1 respectively (all *P* < 0.001 vs untreated control cells). Strikingly, in BxPC-3 and CFPAC-1, 4 h IFN-β pre-treatment resulted already at low concentrations gemcitabine (up to 0.1 ng/ml) in a significant higher cell growth inhibition (1.9- and 1.8-fold lower EC_50_ value respectively, both *p* < 0.001 vs untreated control cells) (Fig. 1d and Fig. S2).

An additional experiment was performed, in which cells were treated with simultaneously 100 IU/ml (BxPC-3 and CFPAC-1) or 1000 IU/ml (Panc-1) IFN-β plus increasing concentrations (0.5–5 ng/ml) gemcitabine for 3 days. In this experimental setting, the overall response of gemcitabine was not significantly stronger compared to cells without IFN-β treatment (Fig. S3).

### IFN-β potentiates the anti-tumor effects of gemcitabine on colony formation

Effect on mean colony size and surviving fraction was assessed after 72 h IFN-β (100 IU/ml for BxPC-3 and CFPAC-1; 1000 IU/ml for Panc-1), after 72 h gemcitabine, and after 72 h IFN-β pre-treatment followed by 72 h gemcitabine monotherapy. Two concentrations gemcitabine were used, in which a minimal effect on cell growth was observed at monolayer culture (1 and 2.5 ng/ml for BxPC-3 and Panc-1; 0.1 and 1 ng/ml for CFPAC-1). PE (%) for BxPC-3, CFPAC-1, and Panc-1 were 19 ± 2.8, 78 ± 18, and 24 ± 7 respectively.

**Fig. 2:**
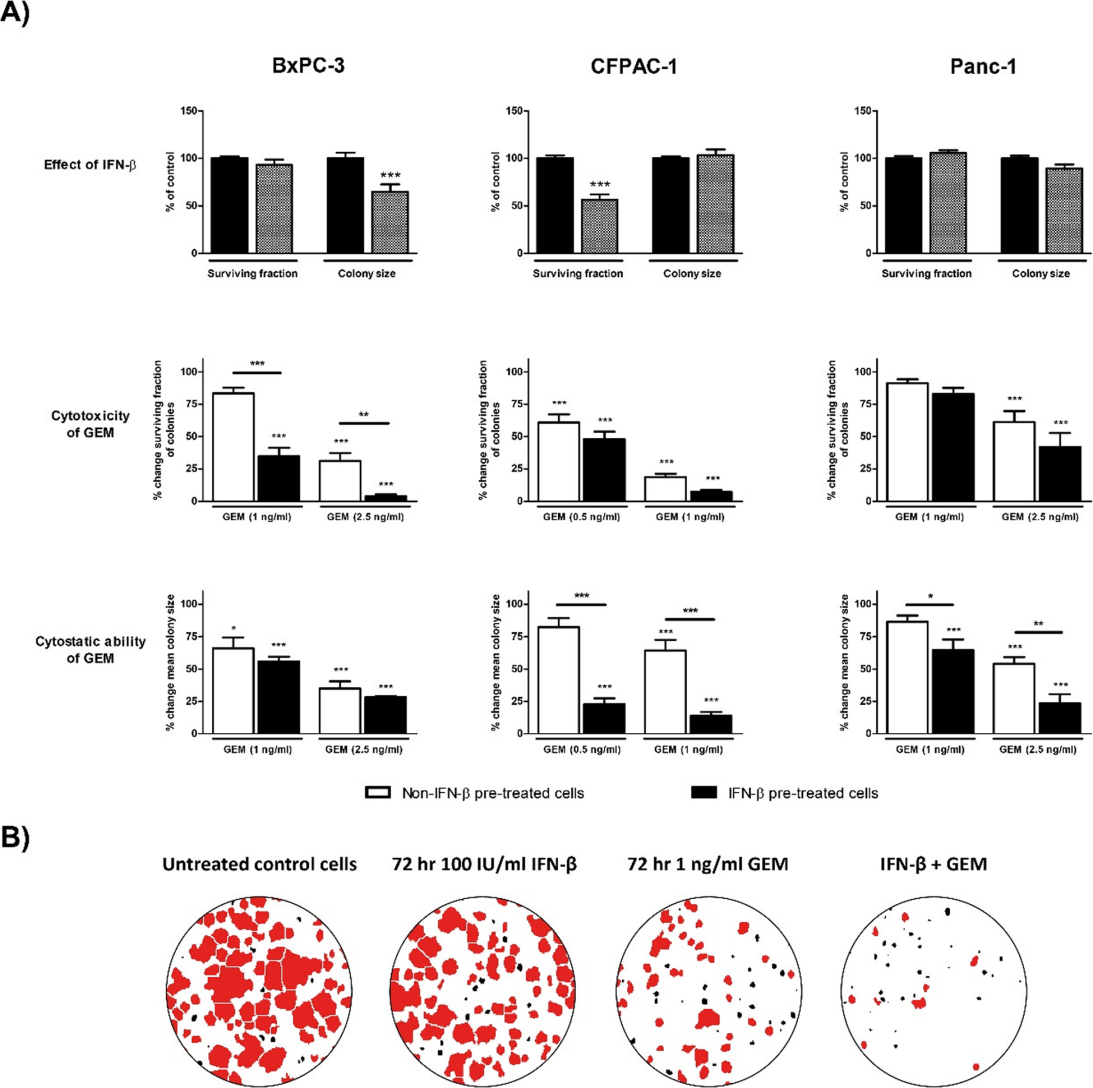
Colony-forming assay. **a** Cytostatic and cytotoxic analysis of colonies in BxPC-3 (left panel), CFPAC-1 (middle panel), and Panc-1 (right panel). Upper panel represents the effect of 72 h IFN-β monotherapy on surviving fraction and colony size. Middle and lower panel represent the effect of 72 h gemcitabine (GEM) in untreated control cells (white bar) versus 72 h IFN-β pre-treated cells (black bar) on surviving fraction and colony size. Used concentrations IFN-β: 100 IU/ml for BxPC-3 and CFPAC-1, and 1000 IU/ml for Panc-1. Used concentrations gemcitabine: 0.5–1 ng/ml for CFPAC-1; and 1–2.5 ng/ml for BxCP-3 and Panc-1. Data are presented as percentage of vehicle treated control. For IFN-β pre-treated cells, effect of IFN-β was set on 100% and used as control. **b** Photomicrographs of treatment effects on BxPC-3 colonies. Red stained colonies represent the measured colonies. Based on cut-off values for number and size, black stained colonies were excluded. Values represent mean ± SEM of at least two independent experiments and are shown as a percentage of control. *p < 0.05, **p < 0.01, and ***p < 0.001 versus control

IFN-β reduced the mean colony size in BxPC-3 and lowered the survival fraction in CFPAC-1 (40 and 44% respectively, both *p* < 0.001). While no effect on survival fraction was observed, IFN-β pre-treatment enhanced the cytotoxic effect of gemcitabine in BxPC-3 (*p* < 0.001). Moreover, the cytostatic effect of gemcitabine was increased in CFPAC-1 and Panc-1 due to IFN-β pre-treatment (both *p* < 0.001) (Fig. 2).

### IFN-β accumulates the proportion of cells in the S-phase

Cell cycle analysis was performed after 72 h IFN-β (100 IU/ml for BxPC-3 and CFPAC-1; 1000 IU/ml for Panc-1), after 72 h gemcitabine (1 ng/ml for CFPAC-1; 2.5 ng/ml for BxPC-3 and Panc-1), and after 72 h IFN-β pre-treatment followed by 72 h gemcitabine monotherapy.

**Fig. 3:**
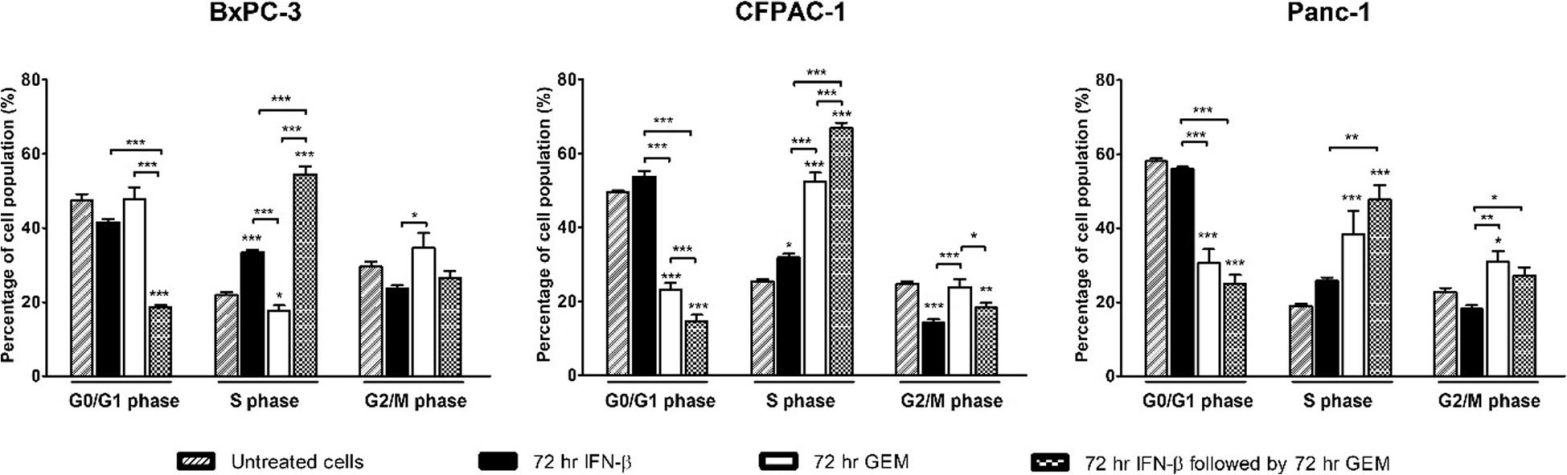
Cell cycle analysis. Cell cycle distribution in BxPC-3 (left panel), CFPAC-1 (middle panel), and Panc-1 (right panel) after 72 h IFN-β, 72 h gemcitabine, and after 72 h IFN-β pre-treatment followed by 72 h gemcitabine monotherapy. Used concentrations IFN-β: 100 IU/ml for BxPC-3 and CFPAC-1, and 1000 IU/ml for Panc-1. Used concentrations gemcitabine: 1 ng/ml for CFPAC-1 and 2.5 ng/ml for BxPC-3 and Panc-1. Values represent mean ± SEM of at least two independent experiments and are shown as relative percentage of the total cell population. *p < 0.05, **p < 0.01, and ***p < 0.001 versus control

IFN-β increased the percentage of cells in the S-phase in BxCP-3 (12%, *p* < 0.001) and CFPAC-1 (7%, *p* < 0.05) (Fig. 3). Additionally, an increase of S-phase population was observed after gemcitabine treatment in CFPAC-1 and Panc-1, which was associated with a decrease of cells in the G_0_/G1-phase (27 and 19% respectively, both *p* < 0.001). In BxPC-3, treatment with gemcitabine caused a minimal decrease of cells in the S-phase (− 4%, *p* < 0.01).

IFN-β pre-treatment followed by gemcitabine resulted in the strongest S-phase accumulation, with an increase of 33, 42, and 29% in BxPC-3, CFPAC-1, and Panc-1, respectively (all *p* < 0.001). This S-phase accumulation was associated with a strong decrease of cells in the G_0_/G_1−_phase (BxPC-3, 28%; CFPAC-1, 35%; Panc-1, 33%; all *p* < 0.001).

### IFN-β upregulates expression of transporter genes involving the intracellular uptake of gemcitabine

**Fig. 4:**
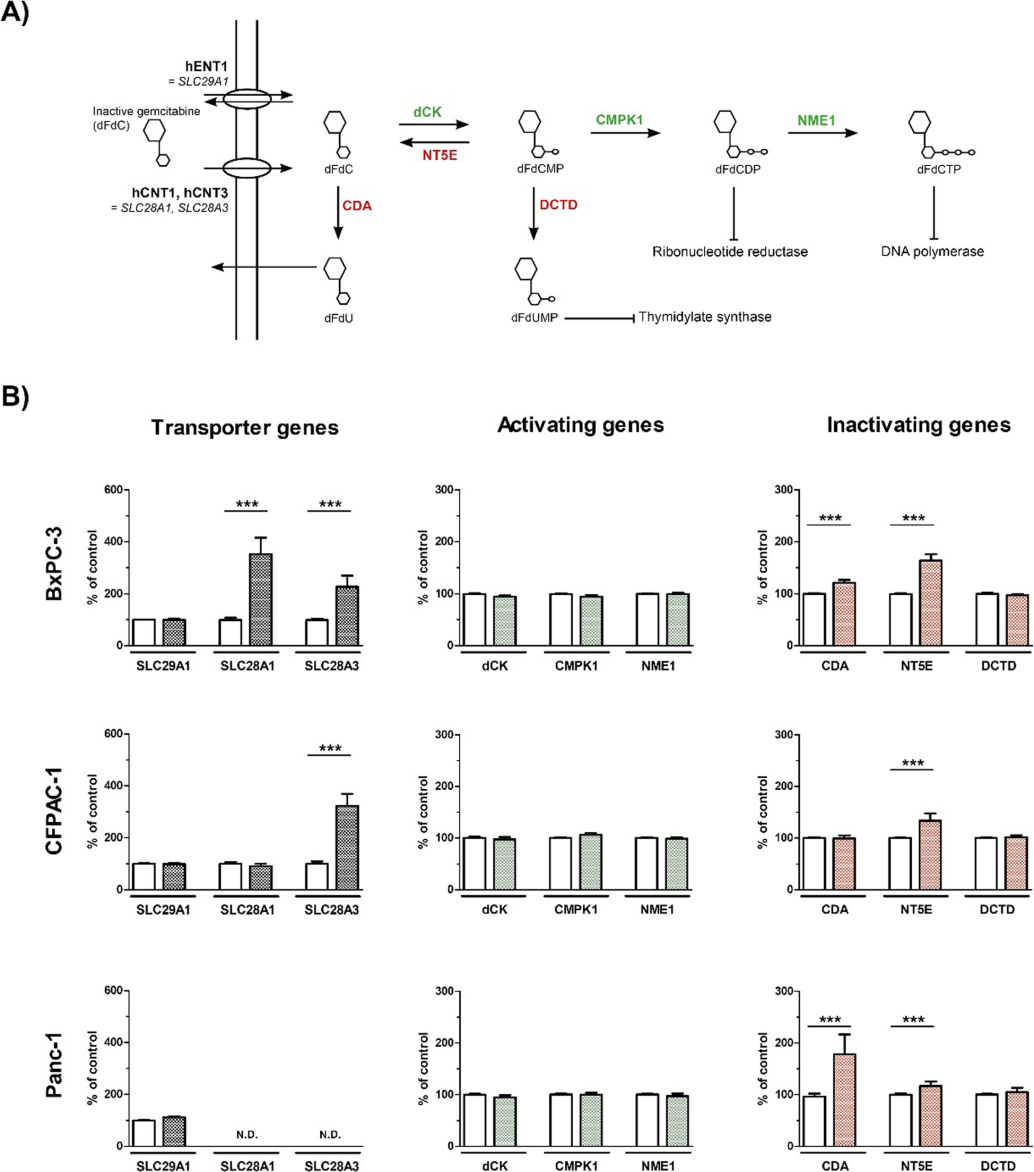
mRNA expression of genes involved in transport and metabolism of gemcitabine in BxPC-3 (upper panel), CFPAC-1 (middle panel), and Panc-1 (lower panel). **a** Schematic overview of the genes encoding for the transporters (*SLC29A1*, *SLC28A1*, and *SLC28A3*), activating enzymes (*dCK*, *CMPK1*, and *NME1*), and inactivating enzymes (*CDA*, *NT5E*, and *DCTD*) of gemcitabine. **b** Percentage change in mRNA expression between untreated control cells (white bars) and after 72 h IFN-β (coloured bars). Used concentrations IFN-β: 100 IU/ml for BxPC-3 and CFPAC-1, and 1000 IU/ml for Panc-1. Values represent mean ± SEM of at least two independent experiments in quadruplicate and are shown as a percentage of control. **p < 0.01 and ***p < 0.001 versus control

As illustrated in Fig. 4a, several genes are involved in the metabolic pathway of gemcitabine. Gene expression analysis was performed of all genes after 72 h IFN-β (100 IU/ml for BxPC-3 and CFPAC-1; 1000 IU/ml for Panc-1). Baseline expression of *SLC28A1* and *SLC28A3* was low (< 0.0001) in BxPC-3 and CFPAC-1, and not detectable in Panc-1 (Fig. S3). IFN-β strongly increased *SLC28A1* and *SLC28A3* in BxPC-3 by 252 and 127%, respectively (both *p* < 0.001). In CFPAC-1, *SLC28A3* was upregulated by 223% (*p* < 0.001). Remarkably, no increase in the expression of these genes was observed in Panc-1. Regarding the inactivating genes, IFN-β increased expression of *CDA* in BxPC-3 and Panc-1 (21 and 78% respectively, both *p* < 0.001). Expression of *NT5E* was increased in all cell lines (BxPC-3, 64% *p* < 0.001; CFPAC-1, 34% *p* < 0.001; Panc-1, 17% *p* < 0.01). No difference in expression of the activating genes was observed (Fig. 4b).

### Ex vivo prevalidation of IFN-β mono- and combination therapy

**Fig. 5:**
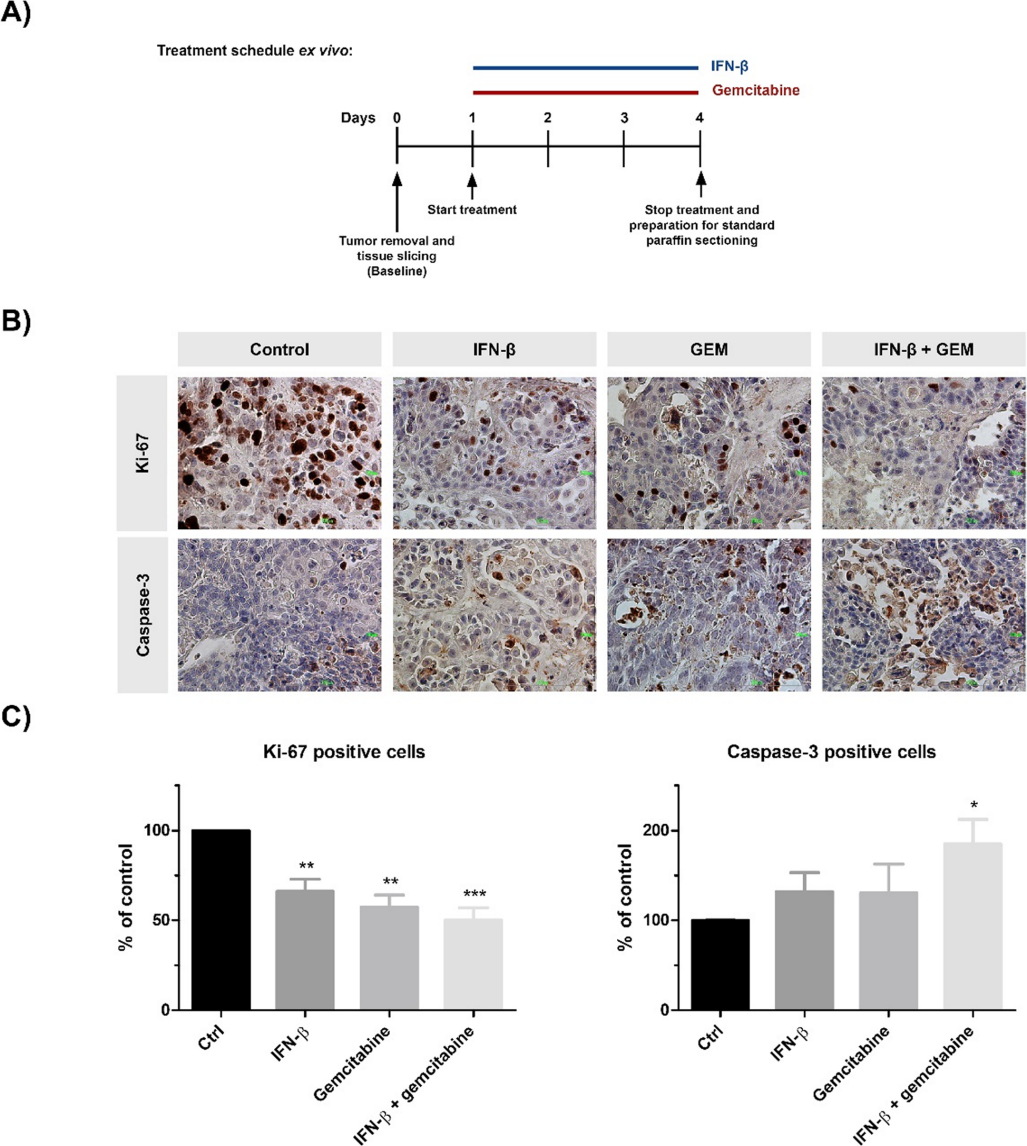
Effects of IFN-β and gemcitabine using an ex vivo precision cut tissue slice model. **a** Experimental design for ex vivo experiments. Slices, derived from xenograft BxPC-3 tumors of untreated mice, were incubated for 72 h without or with IFN-β (100 IU/ml), gemcitabine (1 ng/ml), or the combination of IFN-β plus gemcitabine. **b** Representative tissue slides of human pancreatic cancer xenograft tissue slices stained for KI-67 (upper panel) or caspase-3 (lower panel) in control and treated slices. **c** Immunohistochemical analysis of Ki-67 (left) and cleaved caspase-3 (right) expression, representing the proportion of proliferating cells and the proportion of apoptotic cells respectively. Values represent mean ± SEM of at least three different areas within the tumor and are shown as a percentage of control. **p < 0.01 and ***p < 0.001 versus control

In order to attempt to use a model that might predict treatment effects in vivo, an ex vivo precision cut tissue slice model was used. Four xenograft tumors of untreated mice were used to create the tissue slices. Slices were incubated for 72 h with 100 IU/ml IFN-β, 1 ng/ml gemcitabine, or the combination of IFN-β plus gemcitabine (Fig. 5a).

We next examined the expression of the cell proliferation marker Ki-67 and the apoptosis maker Caspase-3 in tumor tissue slices. The proportion of Ki-67 positive cells was significantly reduced after IFN-β, gemcitabine, and after the combination of IFN-β plus gemcitabine (decrease of 34, 43, and 50% respectively; all *P* < 0.01). Additionally, combination therapy significantly increased the number of apoptotic cells (increase of 85%, *P* = 0.02) (Fig. 5b and c).

### In vivo validation of IFN-β mono- and combination therapy using a subcutaneous pancreatic cancer model

**Fig. 6:**
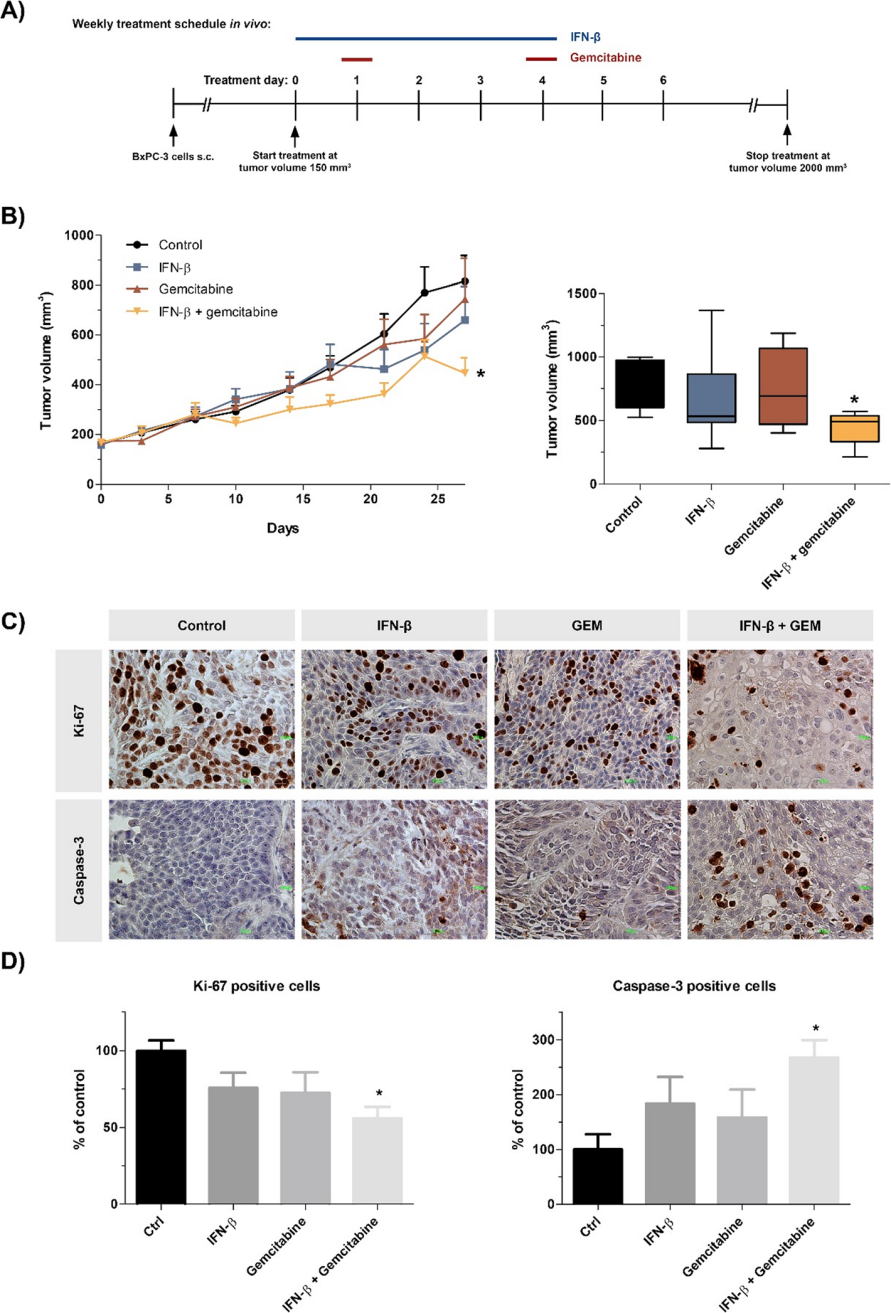
Treatment effects of IFN-β and gemcitabine in a subcutaneous heterotopic human pancreatic cancer model. **a** Experimental design for in vivo experiments. BxPC-3 human pancreatic cancer cells (1 × 10^6^ /100 μl PBS) were subcutaneously injected in nude mice. Seven day later, groups of mice received five times a week, on consecutive days, an i.p. injection of IFN-β (1.5 × 10^5^ IU), two times a week (at day 2 and 4) an i.p. injection of gemcitabine (40 mg/kg), or the combination of IFN-β plus gemcitabine. Mice in the control group received five times a week, on consecutive days, an intraperitoneal (i.p.) injection of 100 μl of 0.9% NaCL. **b** Time course of change in tumor volume (left). After 4 weeks of treatment, mice were sacrificed and tumor volume was measured (right). **c** Photomicrographs of representative tissue slides of immunohistochemical staining for Ki-67 or cleaved caspase-3, **d** Immunohistochemical analysis of Ki-67 (left) and cleaved caspase-3 (right) expression, representing the proportion of proliferating cells and the proportion of apoptotic cells respectively. Values represent mean ± SEM of at least three different areas within the tumor and are shown as a percentage of control. **p < 0.01 and ***p < 0.001 versus control

Finally, we examined the effects of IFN-β and gemcitabine, alone or in combination, on the growth of pancreatic tumors in nude mice. BxPC-3 cells were subcutaneously injected into male Balb/C mice. After 1 week, mice were randomized into one of four treatment arms: vehicle (NaCl), IFN-β (1.5 × 10^5^ IU), gemcitabine (40 mg/kg) or both agents combined (Fig. 6a).

The time course of the growth of tumor volume is depicted in Fig. 6b. After 4 weeks of treatment, a significant reduction in tumor volume by 45% was found compared to untreated mice (*P* = 0.01). No significant weight loss was observed in any of the treatment groups, indicating that all the treatments were well tolerated (Table 1). However, in the gemcitabine arm, one mouse was found dead before start of the treatment and one mouse at the end of the total treatment cycle. Due to organ and tumor lysis, necropsy was not possible anymore.

**Table 1:** Treatment of subcutaneous heterotopic human pancreatic BxPC-3 tumors in nude mice

Treatment groups	Completion of treatment	Tumor volume (mm^3^)		Tumor weight (g)		Body weight (g)	
		Mean	Range	Mean	Range	Mean	Range
Control	5/8^a^	816	524–999	596	373–790	26	24–28
IFN-β	7/8^a^	660	281–1370	507	161–852	26	24–28
Gemcitabine	5/7^a + b^	745	402–1187	628	166–1121	25	24–27
IFN-β + gemcitabine	6/8	447^c^	213–572	492	161–958	26	24–28

^a^ Number of mice that completed 4 weeks of treatment. Mice were sacrificed before the end of treatment if the wellbeing of the animal could not be maintained (in all these mice this was due to ulceration of the tumor)

^b^ One mice died before the start of the treatment

^c^ *P* < 0.05 versus control

As observed ex vivo in the tissue slice experiments, the combination of IFN-β plus gemcitabine significantly reduced the proportion of Ki-67 positive cells with 44%, while apoptosis was increased with 168% in tumor tissues compared with control group (both *P* < 0.05) (Fig. 6c and d).

## Discussion

So far, gemcitabine has demonstrated disappointing results in patients with pancreatic cancer. More effective chemotherapeutics like FOLFIRINOX, where chemoresistance also remains a major clinical problem, is associated with frequent dose reductions, treatment-related serious adverse events, and often grade 3–4 infections. Since gemcitabine is much better tolerated with less toxicity compared to the newer chemotherapeutic agents, especially in the elderly pancreatic cancer patients, we aimed to evaluate whether the anti-tumor effect of gemcitabine could be enhanced by IFN-β and to demonstrate the mechanism of action.

First, we studied the effect of IFN-β alone and in combination with gemcitabine in three human pancreatic cancer cell lines: two IFN-β sensitive cell lines (BxPC-3 and CFPAC-1) and an IFN-β insensitive cell line (Panc-1) [16].

IFN-β strongly increased the inhibitory effects of gemcitabine in the IFN-β sensitive cell lines, which was confirmed in colony-forming assay. Both the cytotoxic and cytostatic effects of gemcitabine were significantly enhanced by IFN-β.

The increased chemosensitivity can be explained by two important observations. First, IFN-β increased cell population in the S-phase, suggesting that these cells were not able to transit into the G_2_/M-phase efficiently, and therefore exhibited a prolonged stay in the S-phase. As a result, DNA replication fails and cells can become more vulnerable for gemcitabine treatment. A downregulation and impaired activity of cyclins and cyclin dependent kinases was previously reported after IFN-β treatment, explaining the prolonged stay in the S-phase [23]. The active metabolites of gemcitabine, dFdCDP and dFdCTP, are also known to inhibit DNA synthesis by inhibiting ribonucleotide reductase and by DNA incorporation, respectively. Consequently, a complete inhibition of DNA synthesis is achieved, and apoptosis is induced [7]. Combination therapy with IFN-β and gemcitabine resulted in the strongest induction of cells in the S-phase in the IFN-β sensitive cell lines. In Panc-1, no difference was observed upon IFN-β treatment, which is in line with the absence of the chemosensitising effect in this cell line.

The second observation is the upregulation of gemcitabine transporters by IFN-β, resulting in potentially increased drug influx. A strong correlation was found between treatment resistance and the expression of gemcitabine transporters (hENT1, hCNT1, and hCNT3), activating enzyme (dCK), and inactivating enzyme (CDA) of gemcitabine [6, 7]. It should be emphasized, however, that we studied the effect of IFN-β on mRNA expression of gemcitabine transporters only. Further studies are necessary to demonstrate that IFN-β treatment results in increased intracellular gemcitabine levels and to confirm this mechanism of action by e.g. transporter knockdown. Nevertheless, this is the first study that evaluated the potential interaction between IFN-β and the expression of genes involved in gemcitabine transport and metabolism of gemcitabine. IFN-β strongly increased the expression of genes encoding for the hCNT1 and hCNT3 transporter in BxPC-3 and CFPAC-1. In contrast, IFN-β upregulated expression of *CDA* in Panc-1 cells.

The time of incubation with IFN-β appeared to be a significant parameter in our study. First, the (chemosensitising) anti-tumor effect was time-dependent. Notably, a 4 h pre-treatment with IFN-β already increased the response to gemcitabine in the IFN-β sensitive cell lines. In line with this, expression of ISGs was upregulated after 4 h and increased over time. Secondly, IFN-β did not enhance the gemcitabine response when both drugs were given simultaneously, suggesting that IFN-β needs time to sensitize tumor cells for chemotherapy.

Remarkably, there were significant differences between the IFN-β sensitive cell lines BxPC-3 and CFPAC-1 and the relative insensitive cell line Panc-1. First, the anti-tumor effects of IFN-β were less pronounced in Panc-1, even though these cells were treated with a 10-fold higher concentration (1000 IU/ml). Thereby, IFN-β pre-treatment mainly resulted in an additive anti-tumor effect in monolayer culture. Surprisingly, while IFN-β monotherapy had no effect on the colony size, it significantly enhanced the effect of gemcitabine on the colony size, suggesting a synergistic effect.

So far, there are no biomarkers for monitoring IFN activity and predicting clinical efficacy during IFN-β treatment. Booy et al. studied the correlation between the expression of the type I interferon receptor and the anti-tumor effect in a large panel of human pancreatic cancer cells. Despite the variable receptor expression among the cell lines, no significant correlation was reported regarding the maximal inhibitory effect of IFN-β [16]. Potentially, the downstream pathway of IFN can predict the response toward IFN-β treatment. In the current study, we measured the expression level of three ISGs (*Mx1*, *IFIT1*, and *OAS1A*), which are the functional end products of the IFN signalling pathway [24]. Therefore, their expression is induced as a result of an active IFN pathway. Consequently, expression levels of these ISGs can be assumed as a representation of activeness of the IFN pathway. At baseline, highest expression was observed in BxPC-3 and CFPAC-1, which is in line with the IFN-β sensitivity. IFN-β upregulated expression of these ISGs in all three cell lines. However, a much stronger upregulation was observed in BxPC-3 and CFPAC-1 compared to Panc-1, suggesting a less active IFN pathway in Panc-1, which might explain the lower response to IFN-β.

Expression of *Mx1* was significant higher (approximately 13-fold) in the IFN-β sensitive cell lines compared to Panc-1, and strongly increased upon IFN-β treatment. The *Mx1* gene encodes for the myxovirus resistant protein A (MxA) protein, which is an important antiviral factor against a wide spectrum of RNA viruses [25]. Apart from its role as a prominent antiviral protein in innate immunity, studies have indicated a role for *Mx1* as a potential tumor suppressor gene. For example, deletion of *Mx1* in prostate cancer is associated with a higher aggressive tendency and the expression of MxA is suppressed in a highly metastatic human prostate carcinoma cell line [26, 27]. Importantly, MxA is also employed to predict the efficacy of chemotherapy in several cancers. Knockout of *Mx1* in prostate cancer cells resulted in a lower sensitivity to the chemotherapeutic agent docetaxel compared to MxA-positive cells [28]. Additionally, a study by Sistigu et al. reported a benefit of high MxA expression in patients with breast cancer receiving anthracycline-based chemotherapy [29]. Above findings indicate an important role for *Mx1* in predicting the response to IFN-β treatment, as well as the potential chemosensitising effects.

So far, the effects of IFN-β, alone and combined with gemcitabine, have not been studied in animal models. By using a heterotopic subcutaneous pancreatic cancer mouse model, we are the first that confirmed the chemosensitising effect of IFN-β in vivo. Based on the response to IFN-β and the amount of IFN receptors expressed, we used the BxPC-3 cells for in vivo experiments [16].

First, we aimed to predict therapy response before start of treatment in an ex vivo tissue slice model. The advantage of this model is the ability to evaluate multiple treatments in one tumor sample. We were able to maintain viable slices up to 4 days of culture, which is in agreement with findings of two other studies [30, 31]. Promising results were observed as the combination of IFN-β plus gemcitabine significantly reduced the proportion of Ki-67 positive cells, while apoptosis was increased in tumor tissues compared with control group.

Regarding in vivo research, the most frequently used gemcitabine concentration varies between 100 mg/kg and 125 mg/kg [32, 33]. Nevertheless, based on the previously described in vitro findings, we decided to reduce the gemcitabine concentration and used a suboptimal concentration of 40 mg/kg.

As expected, given this suboptimal treatment dose, no significant decrease of tumor volume or weight was found in mice treated with gemcitabine alone. Additionally, despite the potent anti-tumor effects in vitro, no difference was found in mice treated with IFN-β alone, however, there was a clear trend towards a smaller tumor volume. Although IFN-β concentrations were not measured in this study, it may be possible that the circulating concentration of IFN-β was not sufficient. This may be related to the relatively short half-life of IFNs in the circulation [34]. In vitro, the concentration of IFN-β required to reduce cell growth to 50% in a large series of pancreatic cancer cell lines, ranged between 70 and 1000 IU/ml [16]. These concentrations are not easily reached (4–10 IU/ml after four doses of 18 MIU IFN-β at 48-h intervals in serum of human healthy volunteers after s.c. administration) [34]. Furthermore, the anti-tumor activities of type I IFNs can be limited by the activation of several survival pathways, such as the induction of the JAK2/STAT-3 pathway, the activation of nuclear factor kappa-beta (NF-κB) and the increased expression of the epidermal growth factor receptor (EGF-R). This could result in the stimulation of cell proliferation, malignant transformation and invasion, and the inhibition of apoptosis [35, 36].

After 30 days of treatment, we observed a significant synergistic effect of the combined therapy of IFN-β and gemcitabine, which was reflected by the reduction of tumor volume and, additionally, by a decreased proportion of proliferating tumor cells and increased apoptosis, confirming the results observed ex vivo.

Although heterotopic subcutaneous models are often used in cancer research, it is important to evaluate the effects of IFN-β and gemcitabine in an orthotopic model as well. Especially since type I IFNs are known to induce immunoregulatory activities and interact with the tumor microenvironment [37].

The therapeutic effectiveness of type I IFN treatment has been demonstrated in a considerable number of other malignancies, including hematologic tumors, as well as solid tumors. However, despite FDA approvals for recombinant IFN-α in a few cancers, including melanoma and renal cell carcinoma, recombinant IFN-α is not a conventional treatment for these malignancies. Long-term administration is needed to maintain therapeutic efficacy, resulting in high-grade toxicity and significant adverse side effects in patients [38].

On the other hand, IFN-β has emerged as a safer and more potent treatment compared to IFN-α. In addition to pancreatic cancer, IFN-β induced evident anti-tumor and chemosensitising effects pre-clinically in several other cancer types, e.g. hepatocellular carcinoma and breast cancer [39, 40]. Thereby, studies report chemosensitising effects with other chemotherapeutic agents, e.g. 5-FU and cisplatin, as well, suggesting that the sensitising effect of IFN-β is not only limited to gemcitabine [39, 41]. Interesting, the intracellular uptake of these drugs are also mediated by the nucleotide transporter proteins hENT1, hCNT1, and hCNT3 [42, 43].

So far, recombinant IFN-β has not yet been approved for the treatment of any cancer type and has yet to be clinically tested in pancreatic cancer. In addition, it is recommended to study the combination of IFN-β with the new developed chemotherapeutic agents such as FOLFIRINOX and Nab-Paclitaxel as well.

While IFN therapies have been around for a while, new insights in activation of the IFN pathway have resulted in novel IFN-directed cancer treatment strategies. One example is the use of IFN based conjugates, which increase the half-life time of IFN and potentially results into higher concentrations at the tumor site [44]. In addition, the PEGylated form of IFN resulted in a higher serum concentration, requiring lower and less frequent doses compared to the conventional IFNs [45]. The PEGylated form of IFN-α has already proven to be effective in the treatment of melanoma and metastatic renal cell carcinoma patients [46, 47]. Currently, the PEGylated form IFN-β is being tested in a phase III clinical trial (ADVANCE) in patients with multiple sclerosis [48, 49]. Another novel approach is the induction of type I IFN production via activation of the STING and RIG-I pathway [44]. These approaches are currently being tested in clinical trials or are in late pre-clinical development.

Although currently no improvement has been made with immunotherapy in pancreatic cancer, IFN-β might also play a crucial role in new strategies in combination with immunotherapy. Expression of Interferon-stimulated gene 15 (ISG15) is induced by IFN-β and its pathway is highly expressed in various malignancies, including pancreatic ductal adenocarcinoma. Interestingly Burke et al. demonstrated that ISG15 pathway knockdown not only reversed the KRAS-associated phenotypes of pancreatic ductal adenocarcinoma cells, such as increased proliferation and colony formation, but also decreased tumor programmed death ligand-1 (PDL-1) expression leading to increased number of CD8+ tumor-infiltrating lymphocytes [50].

## Conclusions

In conclusion, to the best of our knowledge, this is the first study that determined the effects of IFN-β alone and in combination with gemcitabine on pancreatic cancer in three different experimental models. A synergistic anti-tumor effect of the combination treatment with IFN-β and gemcitabine was observed in vitro, in vivo*,* and ex vivo. These anti-tumor effects were already present at low concentrations of gemcitabine and involve cell cycle modulation and upregulation of gemcitabine transporters genes by IFN-β. In order to demonstrate the potent anti-tumor activities of combined gemcitabine/IFN-β therapy in the clinical setting, prospective studies are necessary.

## Supplementary information

**Additional file 1 Figure S1.** Dose response curves of gemcitabine (A) and interferon-bèta (B) on total DNA amount, as a measure of cell number, in ● BxPC-3, ■ CFPAC-1, and ▲ Panc-1 after 3 days of treatment. **Figure S2.** Effect of interferon-bѐta (IFN-β) pre-treatment on gemcitabine response in BxPC-3 (left panel), CFPAC-1 (middle panel), and Panc-1 (right panel). **Figure S3.** Effect of interferon-bѐta (IFN-β) on gemcitabine response in BxPC-3 (left panel), CFPAC-1 (middle panel), and Panc-1 (right panel). **Figure S4.** Baseline mRNA expression of genes involved in gemcitabine transport and metabolism in BxPC-3 (light grey bar), CFPAC-1 (dark grey bar), and Panc-1 (black bar). **Table S1.** Primers and probes used for real time quantitative PCR.

## Data Availability

The datasets used and/or analysed during the current study are available from the corresponding author on reasonable request.

## Abbreviations

dFdC: 2′,2′-difluoro 2′-deoxycytidine
dFdU: 2′2’ difluorodeoxyuridine
β-actin: Bèta-actin
CDA: Cytidine deaminase
CMPK1: Cytidine monophosphate kinase 1
dCK: Deoxycytidine kinase
DCTD: Deoxycytidylate deaminase
EF: Efficiency factor
EGF-R: Epidermal growth factor receptor
GEM: Gemcitabine
GUSB: Glucuronidase bèta
EC50: Half maximal effective concentration
hCNT1: Human concentrative nucleoside transporters 1
hCNT3: Human concentrative nucleoside transporters 3
hENT1: Human equilibrative nucleoside transporter 1
HPRT1: 7 hypoxanthine-guanine phosphoribosyl transferase 1
IFN-β: Interferon-beta
IFIT1: Interferon induced protein with tetratricopeptide repeats 1
ISGs: Interferon stimulated genes
i.p.: Intraperitoneal
Mx1: MX dynamin like GTPase 1
MxA: Myxovirus resistant protein A
NF-κB: Nuclear factor kappa-beta
NME1: Nucleoside diphosphate kinase A
NT5E: 5′-nucleotidases
OAS1A: 2′-5′ oligoadenylate synthetase 1a
PE: Plating efficiency
SLC28A1: Solute carrier family 28 member 1
SLC28A3: Solute carrier family 28 member 3
SLC29A1: Solute carrier family 29 member 1
