# Supplementary material for: Interferon-beta enhances sensitivity to gemcitabine in pancreatic cancer
Source: BMC Cancer. 2020 Sep 23;20:913. doi: 10.1186/s12885-020-07420-0 (PMC7513525; doi:10.1186/s12885-020-07420-0)
Supplement: Supplementary file 1 — Additional file 1 Figure S1. Dose response curves of gemcitabine (A) and interferon-bèta (B) on total DNA amount, as a measure of cell number, in ● BxPC-3, ■ CFPAC-1, and ▲ Panc-1 after 3 days of treatment. Figure S2. Effect of interferon-bѐta (IFN-β) pre-treatment on gemcitabine response in BxPC-3 (left panel), CFPAC-1 (middle panel), and Panc-1 (right panel). Figure S3. Effect of interferon-bѐta (IFN-β) on gemcitabine response in BxPC-3 (left panel), CFPAC-1 (middle panel), and Panc-1 (right panel). Figure S4. Baseline mRNA expression of genes involved in gemcitabine transport and metabolism in BxPC-3 (light grey bar), CFPAC-1 (dark grey bar), and Panc-1 (black bar). Table S1. Primers and probes used for real time quantitative PCR. [file 12885_2020_7420_MOESM1_ESM.docx]

**Supplementary Figures**

**
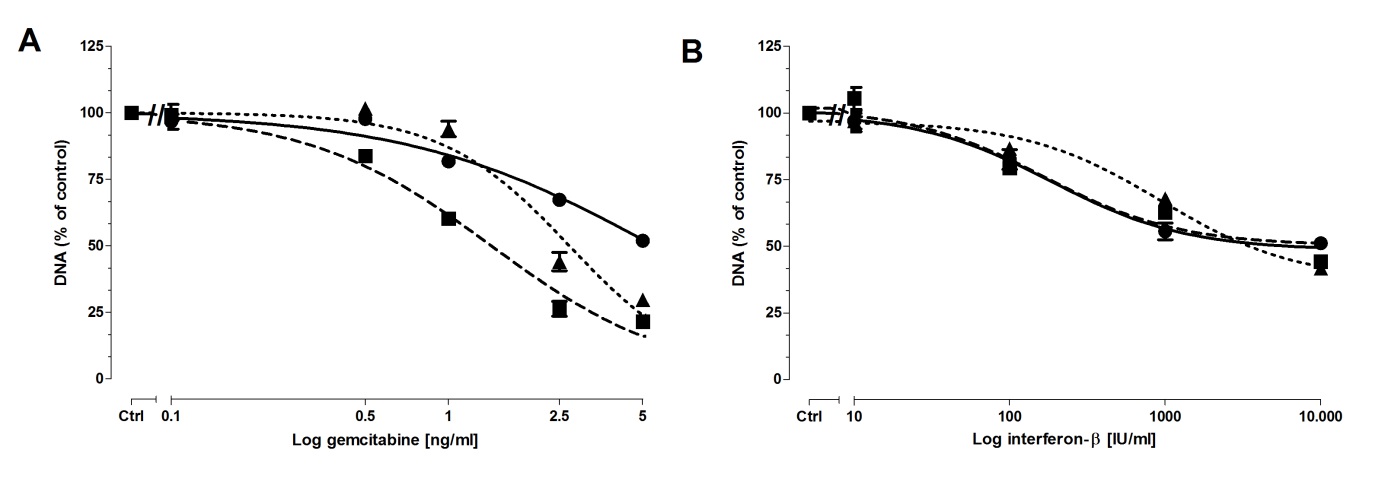
**

**Supplementary Figure 1. Dose response curves of gemcitabine (A) and interferon-bèta (B) on total DNA amount, as a measure of cell number, in ● BxPC-3,** ■ **CFPAC-1, and** ▲ **Panc-1 after 3 days of treatment.** Values represent mean ± SEM of at least two independent experiments in quadruplicate and are shown as the percentage of control.


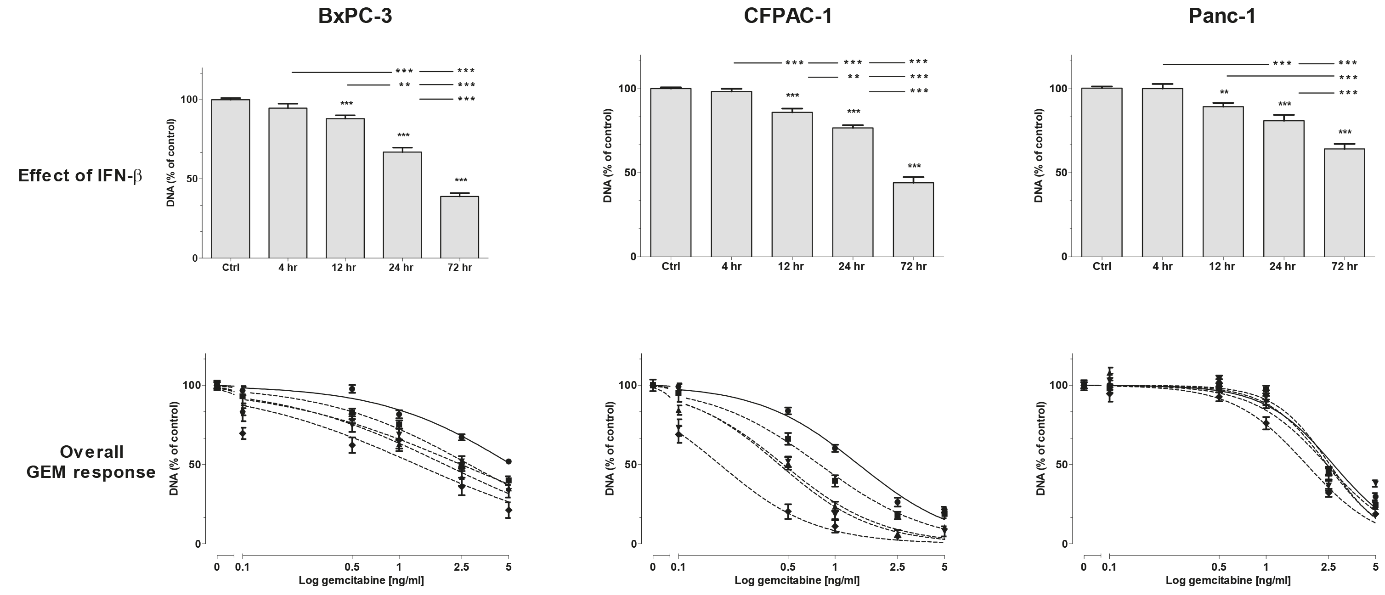


**Supplementary Figure 2. Effect of interferon-bѐta (IFN-β) pre-treatment on gemcitabine response in BxPC-3 (left panel), CFPAC-1 (middle panel), and Panc-1 (right panel).** Upper panel represents the anti-proliferative effect of 4, 12, 24, and 72 hr IFN-β monotherapy. Lower panel represents overall gemcitabine response in untreated control cells (solid line; ●) versus IFN-β pre-treated cells (dotted lines; ■ 4 hr, ▲ 12 hr, ▼ 24 hr, and ♦ 72 hr). Data are presented as percentage of vehicle treated control. For IFN-β pre-treated cells, effect of IFN-β was set at 100% and used as control. Used concentrations IFN-β: 100 IU/ml for BxPC-3 and CFPAC-1, and 1000 IU/ml for Panc-1. Values represent mean ± SEM of at least two independent experiments in quadruplicate and are shown as a percentage of control. **p<0.01 and ***p<0.001 versus control.


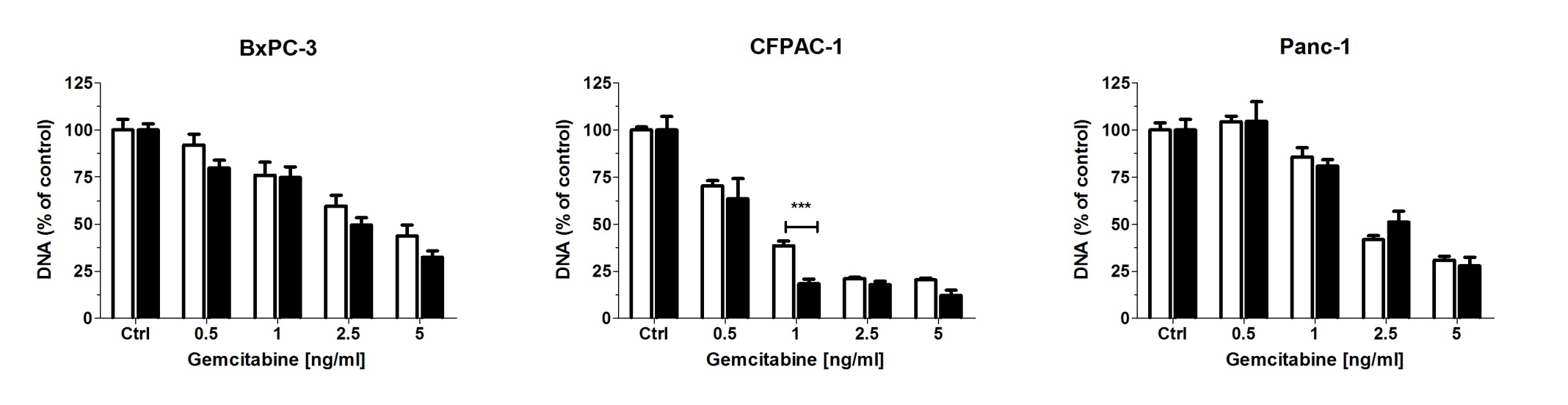


**Supplementary Figure 3. Effect of interferon-bѐta (IFN-β) on gemcitabine response in BxPC-3 (left panel), CFPAC-1 (middle panel), and Panc-1 (right panel).** Ce**l**ls were treated for 3 days with gemcitabine monotherapy (white bars) or with simultaneously IFN-β plus gemcitabine (black bars). Data are presented as percentage of vehicle treated control. For IFN-β treated cells, effect of IFN-β was set at 100% and used as control. Used concentrations IFN-β: 100 IU/ml for BxPC-3 and CFPAC-1, and 1000 IU/ml for Panc-1. Values represent mean ± SEM of at least two independent experiments in quadruplicate and are shown as a percentage of control. ***p<0.001 versus control.


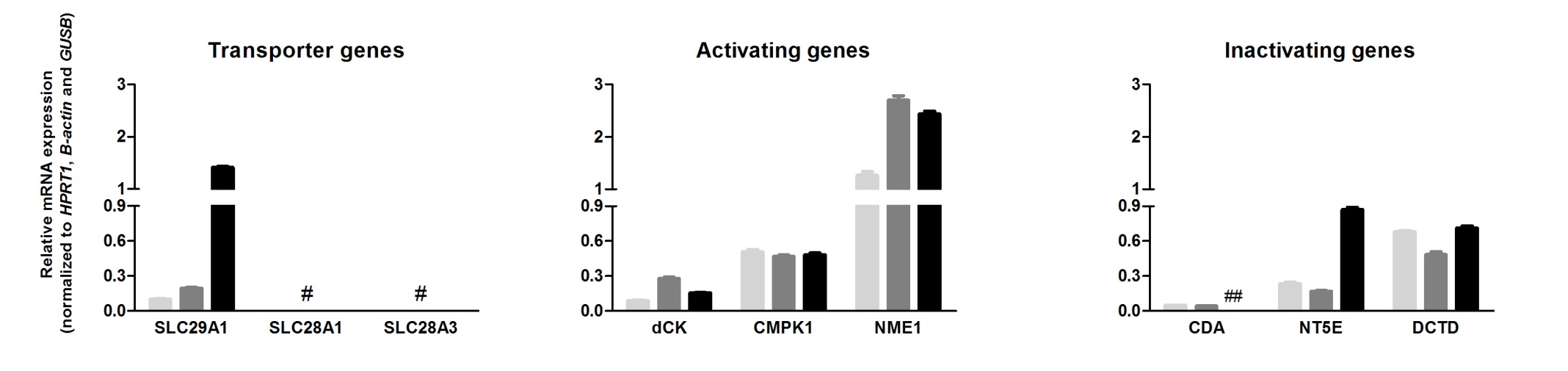


**Supplementary Figure 4. Baseline mRNA expression of genes involved in gemcitabine transport and metabolism in BxPC-3 (light grey bar), CFPAC-1 (dark grey bar), and Panc-1 (black bar).** ^#^ *SLC28A1* and *SLC28A3* expression levels were low (<0.001) in BxPC-3 and CFPAC-1, and undetectable in Panc-1. ^##^ *CDA* expression in Panc-1 was <0.001

**Supplementary Table**

| **Gene** | **Assay ID** | **EF** | |
| --- | --- | --- | --- |
| *HPRT1* | Hs02800695_m1 | 1.97 |  |
| *B-actin* | Hs01060665_g1 | 1.96 |  |
| *GUSB* | Hs00939627_m1 | 1.95 |  |
| *IFIT1* | Hs00356631_g1 | 1.94 |  |
| *OAS1A* | Hs00973637_m1 | 2 |  |
| *Mx1* | Hs00895608_m1 | 2 |  |
| *SLC29A1 (=hENT1)* | Hs1085706_m1 | 1.98 |  |
| *SLC28A1 (=hCNT1)* | Hs00984391_m1 | 2 |  |
| *SLC28A3 (=hCNT3)* | Hs00223220_m1 | 2 |  |
| *dCK* | Hs01040726_m1 | 1.99 |  |
| *CMPK1* | Hs01074420_g1 | 1.97 |  |
| *NME1* | Hs00264824_m1 | 1.95 |  |
| *CDA* | Hs00156401_m1 | 1.88 |  |
| *NT5E* | Hs00159686_m1 | 1.94 |  |
| *DCTD* | Hs01126095_m1 | 2 |  |

**Supplementary Table 1. Primers and probes used for real time quantitative PCR.**

All used primers are commercially available (Thermo Fisher Scientific, Breda, the Netherlands). β-actin, Beta-actin; CDA, cytidine deaminase; CMPK1, cytidine monophosphate kinase 1; dCK, deoxycytidine kinase; DCTD, deoxycytidylate deaminase; EF, efficiency factor; GUSB, glucuronidase beta; hCNT1, human concentrative nucleoside transporters 1; hCNT3, human concentrative nucleoside transporters 3; hENT1, human equilibrative nucleoside transporter 1; HPRT1, 7 hypoxanthine-guanine phosphoribosyl transferase 1; IFIT1, interferon induced protein with tetratricopeptide repeats 1; Mx1, MX dynamin like GTPase 1; NME1, nucleoside diphosphate kinase A; NT5E, 5’-nucleotidases; OAS1A, 2′-5′ oligoadenylate synthetase 1a; SLC28A1, solute carrier family 28 member 1; SLC28A3, solute carrier family 28 member 3; SLC29A1, solute carrier family 29 member.
